# Supplementary material for: Cryo-electron microscopy structure and potential enzymatic function of human six-transmembrane epithelial antigen of the prostate 1 (STEAP1)
Source: J Biol Chem. 2020 May 14;295(28):9502–12. doi: 10.1074/jbc.RA120.013690 (PMC7363144; doi:10.1074/jbc.RA120.013690)
Supplement: Supporting Information [file supp_295_28_9502__index.html]

Cryo-EM structure and potential enzymatic function of human six-transmembrane epithelial antigen of the prostate 1 — Structural insights into human-cancer antigen STEAP1 — Cryo-electron microscopy structure and potential enzymatic function of human six-transmembrane epithelial antigen of the prostate 1 (STEAP1) — Structural insights into human cancer antigen STEAP1 — Supporting Information 

# Cryo-electron microscopy structure and potential enzymatic function of human six-transmembrane epithelial antigen of the prostate 1 (STEAP1)

## Supporting Information

- Supporting Information (to be published online) - Figures S1 - S5, Table S1
